# Immediate Postpartum Copper IUD: A Comparative Analysis between Profiles of Women who Accept and who Refuse it

**DOI:** 10.1055/s-0041-1741452

**Published:** 2022-02-25

**Authors:** Paula Batista Ferreira, Raul Yao Utiyama, Sonia Tamanaha, Erika Tiemi Fukunaga

**Affiliations:** 1 Faculdade de Ciências Médicas, Santa Casa de São Paulo, São Paulo, SP, Brazil

**Keywords:** intrauterine devices, contraception, postpartum period, family planning services, copper intrauterine device, dispositivos intrauterinos, anticoncepção, período pós-parto, planejamento familiar, dispositivo intrauterino de cobre

## Abstract

**Objective** To analyze the profiles of women who accepted and who refused the insertion of the copper intrauterine device (IUD) postpartum and to learn the motivations related to the refusal of the method.

**Methods** Cross-sectional study with 299 pregnant women. The women were informed about the possibility of inserting a copper IUD postpartum and were questioned about their interest in adopting or not this contraceptive. All participants answered a questionnaire with information relevant to the proposals of the present study. The sample size was limited to the number of devices available for the present study.

**Results** A total of 560 women were invited to join the present study and 299 accepted. Out of the 299 women included in the present study, 175 accepted the copper IUD and 124 refused. As the number of pregnancies increased, the IUD acceptance rate raised ( *p* = 0.002), especially between the groups with 1 and with ≥ 4 pregnancies ( *p* = 0.013). Regarding the desire to have more children, the women who planned to have more children were more likely to refuse the method than the ones who did not ( *p* < 0,001).

**Conclusion** Women with multiple pregnancies and desire to not have more children were more likely to accept the copper IUD. The profile of those who refused was first pregnancy and desire to have more children. Among the three most frequent reasons reported for copper IUD rejection, two responses stood out: no specific justification and desire to have more children.

## Resumo

**Objetivo** Analisar o perfil das mulheres que aceitaram e recusaram a inserção do dispositivo intrauterino (DIU) de cobre no pós-parto imediato e conhecer as motivações relacionadas à recusa ao método.

**Métodos** Estudo transversal com 299 gestantes, as quais foram informadas sobre a possibilidade de inserir o DIU de cobre imediatamente após o parto e questionadas sobre o interesse em adotar ou não este contraceptivo. Todas as participantes responderam a um questionário com informações pertinentes às propostas do presente estudo. O tamanho da amostra foi limitado ao número de dispositivos disponíveis para o presente estudo.

**Resultados** Um total de 560 mulheres foram convidadas a participar do estudo, dentre as quais 299 aceitaram. Das 299 participantes, 175 aceitaram o DIU e 124 recusaram. Conforme aumentou o número de gestações, maior foi a taxa de aceitação do DIU ( *p* = 0,002), principalmente quando comparados os grupos de mulheres com 1 e ≥ 4 gestações ( *p* = 0,013). Quanto ao desejo de ter mais filhos, as mulheres que planejavam ter mais filhos tiveram maior chance de recusar o dispositivo do que as que não planejavam ( *p* < 0,001).

**Conclusão** Mulheres com múltiplas gestações e sem desejo de ter mais filhos apresentaram maior probabilidade de aceitar o DIU. O perfil das que recusaram foi primeira gravidez e desejo de ter mais filhos. Dentre os três motivos mais frequentes de rejeição do DIU relatados, duas respostas se destacaram: a falta de justificativa específica e o desejo de ter mais filhos.

## Introduction

The intrauterine device (IUD) is a safe, reversible, and effective contraceptive method, associated with few side effects. 1 2 It is also one of the most used in the world, with a very high percentage of success, with < 1 pregnancy for every 100 women in the 1 ^st^ year of use. 1

Women who want to start contraception in the immediate postpartum period can benefit from the insertion of an IUD to reduce the risks of an unplanned pregnancy and of an undesired short interval between births. 3 4 The IUD is an interesting method for women with difficulty in accessing health services, especially those in situations of social vulnerability and who end up adopting less effective methods. 5 6

In addition, other convenient aspects for inserting the device during this period are the safety of the breastfeeding mother, of the newborn, and the absence of detriment to breastfeeding. 2

Differently from the rest of the world, where 13,9% of women in childbearing age use this method, in Brazil, the IUD is still an underused contraceptive, used by only 3% of women. 7 8 Considering these data, the Municipal Health Department of São Paulo included copper IUDs and hormonal implants in the list of essential drugs. 9

In this context, in 2018, the Obstetrics and Gynecology Department of the Santa Casa de São Paulo implemented the project “Long-term reversible contraception – LARC – in the immediate postpartum period”, with the objective of making this method available and contributing to family planning.

Considering the scarcity of Brazilian studies about acceptance and reasons to refusal of postplacental placement of copper IUD and the high rates of unplanned pregnancies, the objectives of the present study were to analyze the profiles of the women who accepted and who refused the insertion of copper IUDs postpartum and to learn the reasons of refusal, when the offer to insert the IUD was declined.

## Methods

The present cross-sectional study was developed from June 8 to October 8, 2018. During this period, pregnant women admitted to the L&D room of the Santa Casa de São Paulo for childbirth assistance were asked soon upon arriving on the hospital if they had interest in inserting a copper IUD postpartum (within 10 minutes postplacental) and if they would like to take part in the present study. 10

During the study period, 560 pregnant women were admitted to the maternity ward of the Santa Casa de São Paulo for childbirth assistance, and 299 of them accepted to be included in the present study.

The sample size was limited to the number of devices available for the present study (299); therefore, the power of the test was not calculated.

After signing the consent form, all participants answered a questionnaire with relevant information to the proposals of the study and authorized the use of data from their medical records for research. This questionnaire included information on age, marital status, number of pregnancies (number of deliveries, abortions, ectopic pregnancy), number of antenatal consultations, existence of comorbidities or of obstetric complications, previously used contraceptive methods, desire to have more children and, in those who refused to insert a copper IUD, the reason for rejection. The current type of childbirth data was gathered from the medical records.

The exclusion criteria included patients admitted with abortion (defined as termination of pregnancy before 20 weeks or fetal weight < 500 g) and ectopic pregnancy (extrauterine pregnancy), in addition to those who declined to participate in the study. 11 12

The descriptive and comparative analysis of the collected data was made by the Statistics Department of Santa Casa de Sao Paulo School of Medical Sciences using SPSS for Windows version 13.0 (SPSS Inc., Chicago, IL, USA) and Epi Info 3.4.1. The statistical analysis included the calculation of the summary measures such as mean, standard deviation (SD), and minimum and maximum values for continuous variables. Categorical variables were presented as frequency and percentage.

The two groups (women that accepted and women that refused the copper IUD insertion) were compared and, to check if they differed, the t-student test or the Mann-Whitney test for the continuous variables was applied. For categorical variables, the chi-squared test or the Fisher exact test were applied. In all analyzes, the significance level of 5% was adopted.

All participants were informed about the benefits, risks, contraindications, and main adverse effects of the contraceptive offered and accepted to participate voluntarily in the research. The study was approved by the Research Ethics Committee of the Santa Casa de São Paulo.

All devices (TCu 380A IUD, lot 151171, FURP) were offered with no additional cost.

## Results

From the 560 pregnant women who were admitted to the maternity ward of the Hospital da Santa Casa de São Paulo during the study period, 299 (53.4%) accepted to participate in the research and 261 (46.6%) refused. From this group of 299 participants, 175 (58.5%) accepted the insertion of the copper IUD and 124 (41.5%) refused it. Considering all 560 participants, the overall acceptance was 31,25%. The age variation ranged from 15 to 43 years old. The average age of the participants who accepted was 27.9 years old (SD ± 7.2), and that of those who refused the insertion of the copper IUD postpartum was 28.2 years old (SD ± 5.7). Using the t-Student test, a result with no statistic value was obtained ( *p* = 0.768). Therefore, there was no evidence that age increases copper IUD acceptance or rejection. The complete analysis is shown in Table 1 .

**Table 1:** Summary of the results from the analyzed data

Characteristics	Accepted IUD *n* = 175	Refused IUD *n* = 124	*p-value* (chi-squared test)
Marital status – *n* (%)			0.550
Married/Stable union	87 (56.9)	66 (43.1)	
Single	88 (60.3)	58 (39.7)	
Type of childbirth – *n* (%)			0.257
Natural childbirth	117 (60.9)	75 (39.1)	
C-section	58 (54.2)	49 (45.8)	
Number of pregnancies – *n* (%)			0.013
1	47 (49.5)	48 (50.5)	
2	48 (53.9)	41 (46.1)	
3	37 (64.9)	20 (35.1)	
≥ 4	43 (74.1)	15 (25.9)	
Number of antenatal consultations – *n* (%)			0.835
Without consultations	9 (64.3)	5 (35.7)	
< 5	17 (63.0)	10 (37.0)	
≥ 5	148 (58.5)	105 (41.5)	
No information	1 (20.0)	4 (80.0)	
Comorbidities – *n* (%)			0.831
Yes	53 (59.6)	36 (40.4)	
No	117 (58.2)	84 (41.8)	
No information	5 (55.6)	4 (44.4)	
Previous contraceptive method – *n* (%)			0.594
Yes	116 (59.2)	80 (40.8)	
No	33 (47.8)	36 (52.2)	
No information	26 (76.4)	8 (23.6)	
Desire for more children – *n* (%)			< 0.001
Yes	16 (31.4)	35 (68.6)*	
No	94 (71.8)	37 (28.2)*	
Does not know	45 (48.9)	47 (51.1)	
No information	20 (80.0)	5 (20.0)	

Abbreviation: IUD, intrauterine device.

**Fig. 1:**
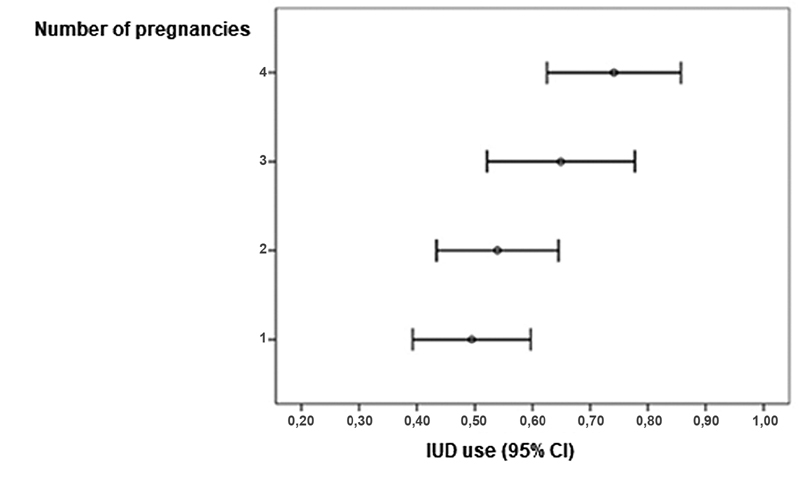
Confidence intervals for copper IUD acceptance in the immediate postpartum period in 299 participants and number of pregnancies.

Marital status and type of childbirth did not influence the decision to insert the copper IUD postpartum ( *p* = 0.550 and 0.257, respectively). Considering the number of pregnancies, it was observed that the higher the number of pregnancies, the higher the copper IUD insertion rate. The result of *p* = 0.002 in the test revealed the statistical difference between the primiparous participants and those with ≥ 4 pregnancies ( Fig. 1 ). This means that multiparity was a factor that increased IUD acceptance in the immediate postpartum period.

**Fig. 2:**
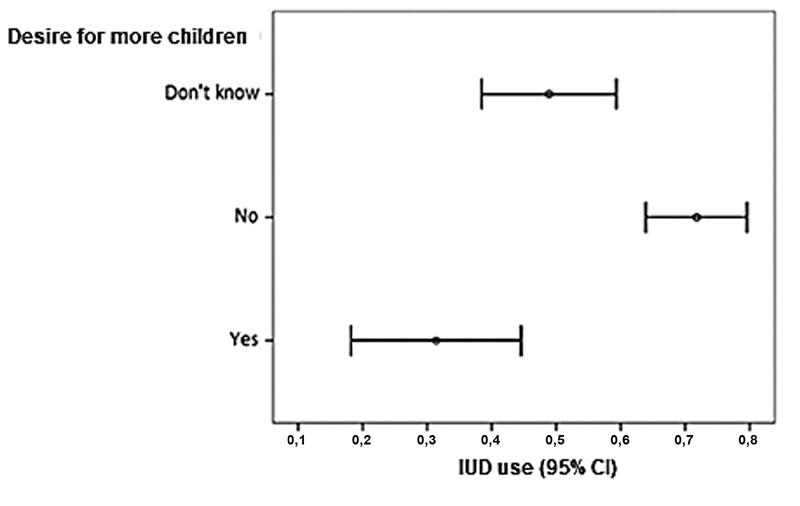
Error bar diagram of desire to have more children and acceptance of the IUD.

On the other hand, the number of antenatal visits, previous comorbidities of the pregnant woman, and use of other contraceptives did not show statistical differences between the groups that accepted and refused the copper IUD in the immediate postpartum period; therefore, these factors did not impact the decision. The present study revealed that for 265 of the 299 participants, the most known and used contraceptive methods were pills (44.2%), condoms (19.2%), other methods (10.6%), and 26.0% of women did not use any method. The rest of the women did not answer this question. The type of contraceptive method used prior to the current pregnancy did not influence the option for the insertion of the IUD in the immediate postpartum period. In contrast, the lack of desire to have more children resulted in greater acceptance of the copper IUD postpartum than in those who intended to have other pregnancies or were in doubt ( Fig. 2 ).

A total of 124 women did not adhere to the method, 106 of whom explained their reason and 18 did not respond (14.5%). Among the 106 women who expressed the reason for rejection, 41 (38.7%) had no specific reason, 25 (23.6%) preferred another contraceptive method, 17 (16%) intended to have more children, 9 (8.5%) had medical contraindications to IUDs, 5 (4.7%) had language barriers, and 9 (8.5%) revealed other reasons.

## Discussion

Actions in the family planning field in Brazil still face many challenges. Despite the existence of several contraceptive methods, unplanned pregnancies and a short period between two pregnancies are still very frequent and expose the lack of effective policies for a significant portion of the population. 13

It is a fact that > 55.4% of Brazilian women who had children did not plan their pregnancy, according to a survey by the National School of Public Health of the Fundação Oswaldo Cruz, which heard 24,000 women between 2011 and 2012. 13

For many women, the hospitalization period for childbirth assistance is the only opportunity for some health system contact, especially in regions where there is still limited access to medical services. Research shows that, on this occasion, patients are more motivated to address the issue of contraception, with the IUD being one of the options that appears, due to its safety, convenience, and few contraindications. 13 14 15

An initiative from the Municipal Health Department of São Paulo to meet this demand was the implementation of the Long-Acting Reversible Contraceptives (LARC) Project in the immediate postpartum and postabortion periods in public service maternity hospitals. 9 Long-acting reversible contraceptives are defined as those that last ≥ 3 years and are represented by subdermal implant and IUD (levonorgestrel intrauterine system and copper IUD). 16

Within this context, since 2018, our institution started offering and making available the copper IUD to pregnant women admitted to the obstetric center, most of them for childbirth assistance. In the case of the T380A copper IUD used in the project, the contraceptive effect lasts for 10 years. It produces an inflammatory, cytotoxic reaction, which is spermicidal, determining endometrial changes, which compromise the quality and viability of sperm. These effects are local and do not interfere with lactation. 17

Unlike short-acting reversible contraceptive methods (administered orally, intramuscularly, vaginally or by transdermal routes), copper IUDs unite high efficacy (both in perfect use and in typical use) and the highest rates of satisfaction and continuity among users. 8 17 A Brazilian study about insertion of IUDs during cesarean section in women without prenatal contraceptive counseling demonstrated that, after 6 weeks, the rate of IUD permanence was 90%, proving it to be an efficient contraception strategy for women, especially those in a situation of social vulnerability. Moreover, the same study found no difference between the expulsion rate during the first 6 weeks and in the period between 6 weeks and 6 months. 18

In addition, the immediate postpartum period portrays an opportune moment to start contraception with a LARC, mainly considering that the rates of missed postpartum consultations are high (10 to 40%), which delays contraceptive guidelines, and the low average time of exclusive breastfeeding in Brazilian puerperal women, of only 50 to 60 days, consequently with early return to fertility. 5 19

In our research, more women accepted the copper IUD postpartum than refused it. However, it is important to remember that 560 women were invited to join the study and that 261 did not want to participate. This nonacceptance may have been a prior intention of not using the proposed method.

From the 299 women who accepted to join the study, 175 (58.5%) participants opted for the insertion of the copper IUD postpartum, and there was greater acceptance of the method by women with a higher number of pregnancies. In contrast, Tang et al. 20 reported that older and multiparous women showed less interest in inserting the IUD postpartum when compared with younger and primiparous women - the justification in his study was the preference for tubal ligation in that group.

It is interesting to note that women who wanted to have more children were more likely to refuse the copper IUD, while those who did not want more children were more willing to accept the device ( Fig. 2 ). We raised the hypothesis of the existence of a misunderstanding about reversibility and the possibility of withdrawing the method when requested, considering that the third most frequent cause of copper IUD refusal was “I want to have more children.” In agreement with our results, Tang et al. 20 reported that a greater interest in the insertion of the IUD postpartum occurred among women who did not wish to have any more children.

Factors such as age, marital status, number of antenatal visits, type of delivery, comorbidities or obstetric complications, and previous use of contraceptive methods did not impact the acceptance or the refusal of the copper IUD postpartum in our study.

In the literature, there is no consensus regarding age. Makins et al. studied the age range and IUD postpartum acceptance in India, Nepal, Sri Lanka, and Tanzania. They observed that, in India, older women were more likely to accept the device, while the same did not happen in the other three countries. 21

Regarding marital status, in some countries, such as Ethiopia and India, there is information that when husbands are against the use of IUDs, there are higher refusal rates among married women. 2 22 In our research, marital status had no statistical significance, but the desire of the husbands was not asked in the questionnaire.

For Gonie et al., 2 the chance of accepting the insertion of the IUD was greater among women who attended more antenatal consultations. In our study, the number of consultations did not interfere with adherence to the method; however, we do not know whether the topic of contraception was addressed during antenatal care or not.

The presence of previous or acquired comorbidities during pregnancy did not prove to be relevant to the decision to insert the IUD immediately after birth. No other studies that correlated these factors were found.

Even though the type of delivery has not shown an association with the desire to the postplacental IUD placement and it was neither pointed out as a reason for rejection of the method, Hochmuller et al. 23 revealed that the type of delivery was a significant predictor for IUD expulsion. Vaginal delivery was 4-fold more likely to be associated with IUD expulsion inserted in the puerperal period than cesarean section. 23 Laporte et al. 24 also found that the odds of IUD expulsion were higher among women with vaginal compared with cesarean delivery.

Hubacher et al., 25 who described similar findings to those of our study, found no relationship between previous use of a particular contraceptive method and adoption of a long-term method after birth.

The three most frequent reasons reported by the participants who refused the copper IUD placement were: no specific reason, preference for another method, and desire to have more children. Our results were different from those described in the Ethiopian study by Gonie et al. 2 In the study performed on the African continent, the three most relevant factors for the refusal of the IUD in the immediate postpartum period were fear of complications, religious beliefs, and refusal of the husband. These factors were not directly questioned to our patients, but they could make up some of the reasons that were not expressed. Possibly, the lack of knowledge about the LARC Project and the moment (immediately after admission to the Obstetric Center) when the IUD was offered may have interfered in this response.

The idea of the ineffectiveness of postpartum IUD was the main reason for rejection in the study by Chacko et al., 26 in which women, consequently, opted for another method. In our study, the second major reason for refusal was the preference for another method; however, none of our patients mentioned the uselessness of the device.

It is worth mentioning that the desire to have more children was the third most frequent cause of refusal in our research, perhaps indicating the belief in the myth that the IUD is a method of permanent contraception, when in fact it is a long-lasting, reversible method that can be removed when desired. 27

Considering all the aspects analyzed, some suggestions to improve adherence to contraception methods would be: training of professionals in Basic Health Units, inclusion of the theme “family planning” in the scope of consultations during antenatal care, and dissemination of the IUD opportunity in the immediate postpartum period.

With these adjustments, the pregnant woman and her partner could research, learn, and reflect more calmly on their contraceptive options and clarify doubts about the different methods, including the copper IUD, especially aspects related to effectiveness, complications such as chronic pain, and reversibility of the method.

We believe that the improvement of the integration and of the alignment of work between the maternity teams and multidisciplinary groups in the Basic Health Units can contribute to more consolidated and assertive decisions by our pregnant women.

The Hospital Central da Santa Casa de São Paulo, since it is a tertiary center, receives referrals from several Basic Primary Health Care Units and serves pregnant women with very different levels of knowledge about the use of the copper IUD, especially immediately after birth. For this reason, we cannot say that the results obtained are extendable to the population as a whole.

The sample size was not calculated with a previous statistical analysis, it was limited to the number of devices available for the study (299) and, therefore, the power of the test was also not calculated. Consequently, the results obtained may not even be extrapolated to the population studied.

The refusal to participate in the study may have occurred due to the prior intentions the pregnant woman of not wanting the method offered, which was not considered in the study.

The IUD was offered after admission to the L&D room and the pregnant women were in different stages of labor, which is as aspect that was not considered in our study and could have influenced the decision.

## Conclusion

Women with multiple pregnancies and desire to not have more children were more likely to accept the copper IUD. The profile of those who refused was first pregnancy and desire to have more children. Among the three most frequently reported reasons for copper IUD rejection, two responses stood out: no specific justification, and desire to have more children. This highlighted the importance and the need to improve previous educational actions on contraception and clarifications on the mechanisms of action and reversibility of this method.
